# The cold-stress responsive gene *DREB1A* involved in low-temperature tolerance in Xinjiang wild walnut

**DOI:** 10.7717/peerj.14021

**Published:** 2022-09-08

**Authors:** Liqun Han, Kai Ma, Yu Zhao, Chuang Mei, Aisajan Mamat, Jixun Wang, Ling Qin, Tianming He

**Affiliations:** 1 College of Horticulture, Xinjiang Agricultural University, Urumqi, China; 2 Institute of Horticultural Crops, Xinjiang Academy of Agricultural Sciences/Key Laboratory of Genome Research and Genetic Improvement of Xinjiang Characteristic Fruits and Vegetables/Xinjiang Fruit Science Experiment Station, Ministry of Agriculture, Urumqi, China; 3 College of Plant Science and Technology, Beijing University of Agriculture, Beijing, China

**Keywords:** Xinjiang wild walnut, *JfDREB1A* gene, Low-temperature stress, Transgenic *Arabidopsis*, Cold resistance

## Abstract

**Background:**

Low-temperatures have the potential to be a serious problem for plants and can negatively affect the normal growth and development of walnuts. DREB1/CBF (Dehydration Responsive Element Binding Protein 1/C-repeat Binding Factor), one of the most direct transcription factors in response to low-temperature stress, may improve the resistance of plants to low-temperatures by regulating their functional genes. However, few studies have been conducted in walnut. The Xinjiang wild walnut is a rare wild plant found in China, with a large number of excellent trait genes, and is hardier than cultivated walnuts in Xinjiang.

**Methods:**

In this work, we identified all of the DREB1 members from the walnut genome and analyzed their expression levels in different tissues and during low-temperature stress on the Xinjiang wild walnut. The *JfDREB1A* gene of the Xinjiang wild walnut was cloned and transformed into *Arabidopsis thaliana* for functional verification.

**Results:**

There were five DREB1 transcription factors in the walnut genome. Among them, the relative expression level of the *DREB1A* gene was significantly higher than other members in the different tissues (root, stem, leaf) and was immediately un-regulated under low-temperature stress. The overexpression of the *JfDREB1A* gene increased the survival rates of transgenic *Arabidopsis* lines, mainly through maintaining the stability of cell membrane, decreasing the electrical conductivity and increasing the activities of antioxidant enzymes including superoxide dismutase (SOD), peroxidase (POD), and catalase (CAT). Additionally, the expression levels of cold-inducible genes like *AtKIN1*, *AtERD10*, *AtRD29A*, * AtCOR15A* and *AtCOR47*, were significantly increased. These results showed that the *JfDREB1A* gene may play an important role in the response to cold stress of the Xinjiang wild walnut. This study contributes to our understanding of the molecular mechanism of the Xinjiang wild walnut’s response to low-temperature stress and will be beneficial for developing walnut cultivars with improved cold resistance.

## Introduction

Low temperatures are an abiotic stress that affect the growth, development, and distribution of plants, and may even lead to plant death (Colinet, Renault & Roussel, 2017; Shi, Ding & Yang, 2018). As global climate change intensifies, extreme climates and low-temperature disasters will occur more frequently. The study of a plant’s response to low-temperature stress is a topic of great interest in the field of plant research and has important theoretical and practical value for ensuring the plant’s sustainable development (Ding, Shi & Yang, 2019; Gong et al., 2020; Wu, Mao & Zhao, 2021).

Through evolution, plants have gradually formed a complex and efficient response mechanism to adjust their phenotypic structure, physiological characteristics, and molecular mechanism to adapt to low-temperature stress. The most intuitive performance is their phenotypic changes, especially morphological characteristics liking leaf wilting, tissue softening and dehydrating, even withering and dying (McCully, Canny & Huang, 2004; Ruelland et al., 2009). The fundamental mechanism of plant cold stress lies in the damage of the membrane system. The cell membrane is the main medium for the exchange between cells and environmental substances, and its stability is an important factor to judge the strength of plant cold stress tolerance (Muramatsu et al., 2015). Physiological indexes can more accurately reflect the response of plants to low-temperature stress, among them relative electric conductivity, superoxide dismutase (SOD) activity, peroxidase (POD) activity, and catalase (CAT) activity are used to identify the response of plants under low-temperature stress (Anwar et al., 2018; Kim & Tai, 2011; Velikova, Yordanov & Edreva, 2000).

Plants can respond to low-temperature stress by regulating gene expressions in the stress signal pathway through transcription factors (Agarwal et al., 2006; Zhu, 2016). DREB are specific transcription factors in the APETALA2/ethylene response factor (AP2/ERF) family, and combine with dehydration responsive element/C-repeat (DRE/CRT) elements for expressing abiotic stresses such as low-temperature, drought and salt (Haake et al., 2002; Magome, Yamaguchi & Hanada, 2004; Shi, Ding & Yang, 2018; Xie et al., 2019; Zhao et al., 2016). DREB transcription factors are divided into six subgroups, A1–A6. DREB1 belongs to the A1 subgroup which contains a plant-specific and highly-conformed AP2 domain consisting of approximately 60 amino acids and two characteristic sequences (PKK/RPAGRTKFRETRHP, DSAWR) (Jaglo et al., 2001; Nakano et al., 2006; Sakuma et al., 2002). *DREB1/CBF* genes have been identified in rice, tomato, poplar, grape and other plants, and played a key role in the response to low-temperatures (Ito et al., 2006; Maestrini et al., 2009; Xiao et al., 2006; Zhang et al., 2004). Among them *DREB1A/CBF3*, *DREB1B/CBF1* and *DREB1C/CBF2* were reported to be sensitive to low-temperature stress (Fowler & Thomashow, 2002; Park et al., 2015; Gilmour, Fowler & Thomashow, 2004; Thomashow, 2010).

Walnut, the genus *Juglans* of Juglandaceae, is one of the four major nuts in the world. Low-temperatures can seriously affect the normal growth and development of the walnut, resulting in a negative economic impact. Therefore, it is very important to study the response mechanism of the walnut to low-temperature stress. Wild plants are important materials from which to obtain resistance genes that have been selected by their exposure to extreme natural environments. Xinjiang wild walnut (*Juglans Fallax* D.) is a rare wild plant resource in China and the direct ancestor of cultivated walnuts in Xinjiang. It has formed many unique variations in the long-term evolution process and has great potential for utilization (Dong et al., 2012). Our team found that there were more cold-resistant varieties in the Xinjiang wild walnut community than cultivated walnuts (Han et al., 2019). It is important to analyze the molecular mechanisms of the Xinjiang wild walnut in response to low-temperature stress and to detect cold resistance candidate genes. Therefore, we identified the all *DREB1* members from the walnut genome (https://www.ncbi.nlm.nih.gov/bioproject/291087), analyzed the expression levels of the *DREB1* genes in different tissues and low-temperature stress, and defined the key gene, *DREB1A,* in the response to low-temperature stress. The *JfDREB1A* gene of the Xinjiang wild walnut was cloned and transformed into *Arabidopsis* to verify functional mechanism. This study may provide an important candidate gene for improving the cold-resistance of walnut by transgene technique.

## Materials & Methods

### Plant materials

The experimental materials were the ‘Microoblonga’ type of the Xinjiang wild walnut, which has been shown to have strong cold resistance. We collected the mature seeds stored sand storage at 4 °C for three months, the seeds were sowed in nutrition bowls (Peat soil: perlite = 3:1) and were irrigated with water, resulting in 100 seedlings. The roots, stems and leaves were gathered when the seedlings grown to five compound leaves. Meanwhile 35 seedlings with consistent growth were selected for 0 h, 1 h, 2 h, 4 h, 8 h, 12 h and 24 h at 4 °C in the dark in the low temperature artificial climate incubator (DLRX-450B-LED, Jinwen, Shanghai, China). Each sample had three replicates, with five seedlings per replicate. We collected two to five leaves under the parietal lobe and the leaves were frozen immediately in liquid nitrogen and stored at −80 °C before RNA extraction.

The Columbia wild-type *Arabidopsis* was used as the receptor material. The transgenic and wild-type *Arabidopsis* were seeded in a MS medium and were cultured at 23 °C for 16/8 h (day/night). The seedlings were divided into two parts: one cultured in medium for 14 days, the other were transferred to the nutrition bowls (Peat soil: vermiculite: perlite = 1:2:0.5) and grew for 21 days which were used for phenotypes, physiological indexes and genes expression analyzing. The sampling method was the same as above.

### Identification of DREB1s transcription factors in walnut

The walnut whole genome sequence (PRJNA291087) was downloaded from NCBI (https://www.ncbi.nlm.nih.gov/bioproject/291087). The AP2 Hidden Markov Model (PF00847) was downloaded from the Pfam (http://pfam.xfam.org) database. The sequences were screened by HMMER3.0 for those containing AP2 domains from the walnut whole genome. The sequence structures were predicted using the SMART (http://smart.embl.de/smart/batch.pl) and ExPASy-PROSITE (https://prosite.expasy.org/), then the partial length and repetitive sequences were removed. All DREB transcription factors of walnut were identified according to the number and characteristic of their AP2 domains (Nakano et al., 2006).

The *Arabidopsis* DREB transcription factors family was downloaded from the *Arabidopsis* genome database (https://www.arabidopsis.org/). We aligned multiple sequences with the DREB transcription factors of walnut and *Arabidopsis* using Clustal W. A phylogenetic tree was constructed using MEGA 7.0 with the neighbor-joining method, which the execution parameters were bootstrap method 1000, Poisson model, and pairwise deletion.

### Real-time quantitative PCR verification

Total RNA was extracted from leaves of Xinjiang wild walnut seedlings using an RNA extraction kit (TIANGEN, Beijing, China), and 1 µg was used to synthesize the cDNA by using the High Capacity cDNA Reverse Transcription kit (Thermo Fisher Scientific, Shanghai, China). Primers were designed with Primer Premier 6.0 software according to the sequence of five *DREB1* genes in walnut, and 18S rRNA was used as the reference gene (Table S1). Amplification was performed using an qTOWER^3^ G fluorescent quantitative PCR instrument (Analytikjena Company, Jena City, Thuringia, Germany) according to the manufacturer’s instructions (SYBR Green qPCR Master Mix kit (Servicebio, Wuhan, China)), which contained 7.5 µL of qPCR mix, 0.75 µL each of forward and reverse primers (2.5 µM), 2.0 µL of cDNA templates and ddH_2_O added to 15 µL. The PCR cycling conditions were: 95 °C for 10 min followed by 40 cycles of 95 °C for 15 s, 60 °C for 30 s, and the melting curve was 65–95 °C for 0.3 °C/15 s. Each sample had three replicates and the relative expression levels were calculated by the method of 2^−ΔΔCt^.

### Gene cloning

The total RNA was extracted and reverse transcription was performed as described above. The primers were designed with Primer Premier 6.0 software according to the *DREB1A* gene sequence in walnut (Table S1). Amplification was performed using a Biometra TAdvanced 96G PCR instrument (Analytikjena Company, Jena City, Thuringia, Germany). The PCR amplification system followed the manufacturer’s instructions (PrimeSTAR GXL Premix (TaKaRa, Dalian, China)) which contained 12.5 µL of PrimeSTAR GXL Premix, 0.5 µL each of forward and reverse primers (10 µM), 1 µL of cDNA templates and ddH_2_O added to 25 µL. The PCR cycling conditions were: 98 °C for 5 min followed by 30 cycles of 98 °C for 10 s, 56 °C for 15 s, 72 °C for 60 s. The amplified products were determined by 1% agarose gel electrophoresis, then purified and recycled using MiniBEST Agarose Gel DNA Extraction Kit Ver.4.0 (TaKaRa, Dalian, China), connected with pMD19-T vector (TaKaRa, Dalian, China), and transformed by the *Escherichia coli* DH5 *α* competent cell. The full-length cDNA sequence of *JfDREB1A* gene was obtained by sequencing.

### Generation of transgenic *Arabidopsis thaliana* lines

The open reading frame (ORF) of the *JfDREB1A* gene was constructed in the pCAMBIA1304 vector (Cambia, Canberra, Australia). The recombinant plasmid was transformed into the *Agrobacterium tumefaciens* GV3101 strain by freezing and thawing, then transformed into *Arabidopsis* by soaking (Ni et al., 2013). Eventually, the T3 generation transgenic *Arabidopsis* lines were obtained through breeding.

### Survival rates of seedlings counting

The petri dish and potted seedlings of transgenic and wild-type *Arabidopsis* with consistent growth were treated at −7 °C in the dark for 5 h, 8 h and 6 h, 9 h respectively in the low temperature artificial climate incubator (DLRX-450B-LED, Jinwen, Shanghai, China), then were recovered after 4 d at 23 °C for 16/8 h (day/night). The survival rates of the seedlings were counted.

### Relative electric conductivity, SOD, POD and CAT measuring

The potted seedlings of transgenic and wild-type *Arabidopsis* were treated at 4 °C in the dark for 0 h, 1 h, 2 h, 4 h, 8 h, 12 h and 24 h. The relative electric conductivity was measured using a conductometer (DDS-307A, REX, China). The SOD activity was measured using nitrogen-blue tetrazole photoreduction, POD activity was measured using guaiacol colorimetry, and the CAT activity was measured using ammonium molybdate colorimetry, all the methods were carried out according to the instructions of the kits (COMIN, Suzhou, China).

### Functional genes verification

The potted seedlings of transgenic and wild-type *Arabidopsis* were treated with 4 °C in the dark for 0 h, 8 h. Total RNA extraction, reverse transcription, and qPCR amplification were performed as described above. Primers were designed according to the sequence of cold-inducible genes, and the *AtUBQ3* gene (NC_003076.8) was used as an internal reference (Table S1). The relative expression levels were calculated by the method of 2^−ΔΔCt^.

### Statistical analysis

The data were analyzed using SPSS 21 software (SPSS, Inc. Chicago, IL, USA) and presented as means ±  standard deviation (SD), one-way analysis of variance (ANOVA), and the Duncan’s multiple comparison tests at a significance level of *p* < 0.05.

## Results

### *DREB1s* genes in walnut

**Figure 1:**
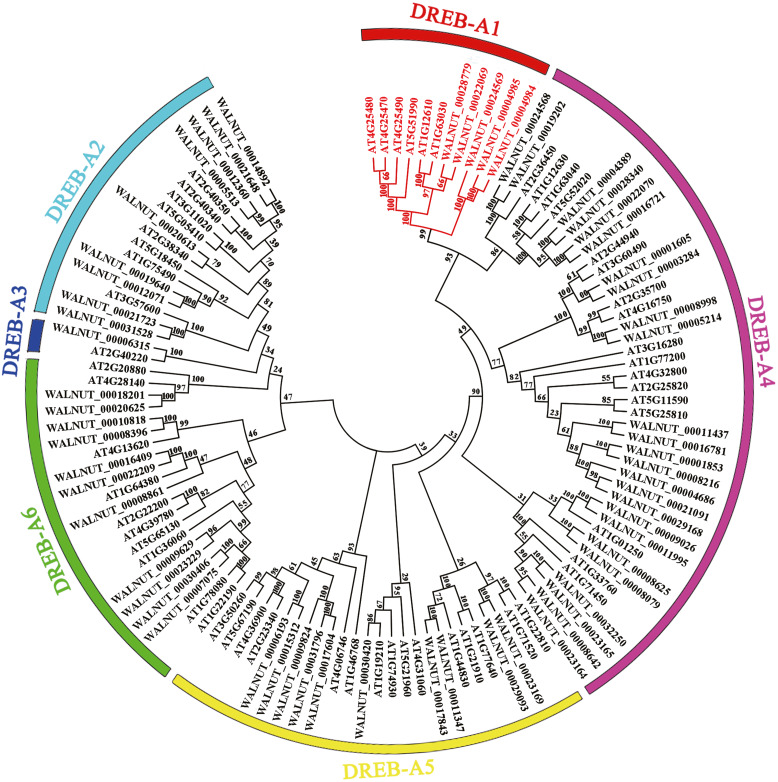
Phylogenetic tree of DREB transcription factor families in walnut and *Arabidopsis thaliana*. Full-length amino acid sequences were aligned using the Clustal W software package. The phylogenetic tree was constructed using the *MEGA 7.0* software package (https://www.megasoftware.net/). The phylogenetic trees were derived using the neighbor-joining (NJ) method with bootstrap value of 1,000 replicates. The tree divided the *DREB1* into six subgroups (A1-A6) represented by different colored clades. The *DREB1* genes are annotated with red font in walnut and *Arabidopsis*.

We found 197 protein sequences containing the AP2 domain from the whole genome database of walnut. A total of 192 protein sequences were identified as being a member of the AP2 family after excluding incomplete and repetitive sequences, with 61 protein sequences of the DREB subfamily (Fig. S1, Tables S2 and S3). Multiple sequence alignment results showed that the DREB subfamily in walnut was divided into six subgroups (A1, A2, A3, A4, A5 and A6). *DREB1* belonged to the A1 subgroup with five genes (*WALNUT_00028779, WALNUT_00022069, WALNUT_00024569, WALNUT_00004985* and *WALNUT_00004984)* (Fig. 1, Table S3). These five genes were homologous to *DREB1A*, *DREB1B*, *DREB1C*, *DREB1Da* and *DREB1Db* in *Arabidopsis,* respectively (Table 1).

**Table 1:** Homologous sequences of five *DREB1* genes related *AtDREB1* in walnut.

NO	Walnut gene	*Arabidopsis thaliana*		Identity	E-value	Score
		Gene name	Gene ID			
1	WALNUT_00028779	*DREB1A*	AT4G25480	55.30	4e−62	192
2	WALNUT_00022069	*DREB1B*	AT4G25490	55.61	1e−63	198
3	WALNUT_00024569	*DREB1C*	AT4G25470	52.69	5e−51	164
4	WALNUT_00004985	*DREB1D*	AT5G51990	39.29	2e−41	444
5	WALNUT_00004984	*DREB1D*	AT5G51990	38.68	7e−44	148

### Expression pattern of *DREB1s* genes

**Figure 2:**
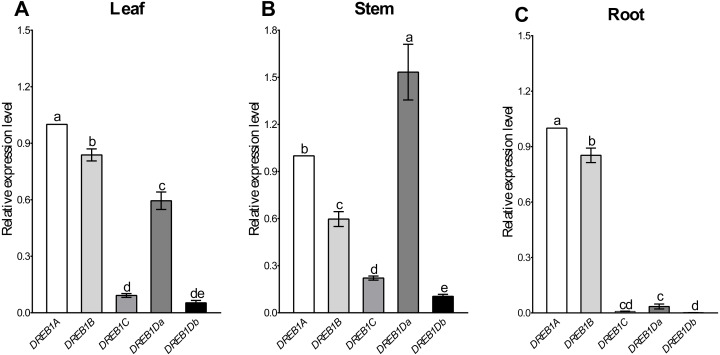
Expression levels of *DREB1* genes in different tissues of Xinjiang wild walnut. (A–C) The expression levels of five *DREB1* genes in the leaf, stem, root of Xinjiang wild walnut seedlings, respectively. Five *DREB1* genes from the walnut genome sequence (PRJNA291087) were identified through bioinformatics methods. The roots, stems and leaves were sampled for expression analysis after the seedlings of the Xinjiang wild walnut grew to five compound leaves. The error bars indicate the SDs from three biological replicates. The different letters above the bars indicate significant differences at the *P* < 0.05 level according to Duncan’s multiple comparison tests.

Tissue-specific expression patterns of the five *DREB1* genes were analyzed using Xinjiang wild walnut seedlings. As shown in Fig. 2, *DREB1A* and *DREB1B* were highly expressed in the root, stem and leaf, among which *DREB1A* was higher, while the expression levels of *DREB1C* and *DREB1Db* were low in these tissues. The expression levels of *DREB1Da* were low in the root, but high in both the stem and the leaf.

**Figure 3:**
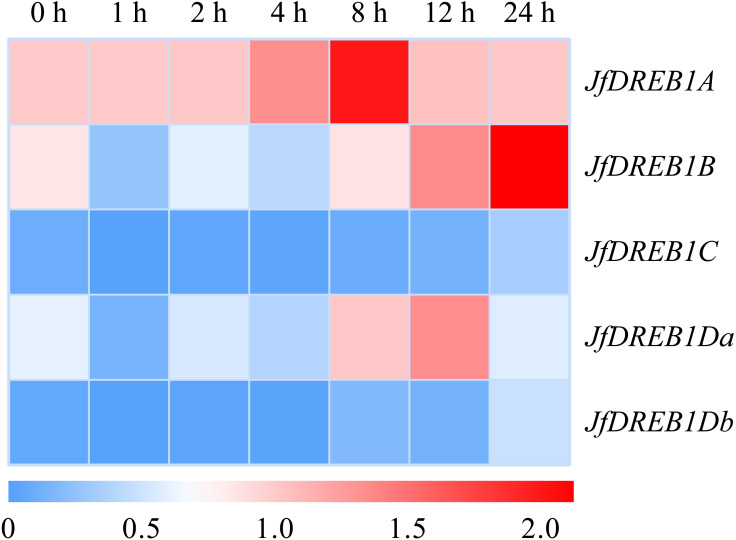
Expression levels of *DREB1* genes in the Xinjiang wild walnut under low temperature stress. The seedlings of the Xinjiang wild walnut with consistent growth were selected for low temperature treatment (4 °C) for 0 h, 1 h, 2 h, 4 h, 8 h, 12 h and 24 h. The leaves under the parietal lobe were used for expression analysis. The vertical axis represents five *JfDREB1* genes, the horizontal axis represents the stage of low temperature stress, red and blue colors in the heat map represent the high and low levels of expression of *JfDREB1s* respectively.

The expression patterns of *DREB1s* genes in the seedlings of Xinjiang wild walnut were determined under low-temperature stress (4 °C) to select for the cold responsive member. The results showed that the expression level of the *DREB1A* gene was up-regulated rapidly 1 h after the low-temperature stress, increased to the highest level at 8 h, then decreased (Fig. 3). While the expression levels of the *DREB1B*, *DREB1Da* and *DREB1Db* genes increased slightly, and fewer folds. The expression levels of the *DREB1C* gene remained lower during this process with no obvious changes (Fig. 3).

### Obtaining the *JfDREB1A* gene from Xinjiang wild walnut

Analysis of the expression levels of the *DREB1s* genes showed that the *DREB1A* gene was significantly higher than other members in the different tissues and was immediately un-regulated under low-temperatures. It can be regarded as a key *DREB1* gene in response to low-temperature stress in walnut. This gene was cloned from the Xinjiang wild walnut and named *JfDREB1A*, with a coding region for 645 bp (Fig. S2, Table S4), showing the structural characteristics of the DREB1/CBF transcription factor (Fig. S3).

### Overexpression of the *JfDREB1A* gene enhanced tolerance of transgenic *Arabidopsis* under low-temperature stress

An overexpression vector was constructed to transform the *JfDREB1A* gene into *Arabidopsis*. The overexpressed T3 generation transgenic homozygous strains were obtained (Fig. S4).

**Figure 4:**
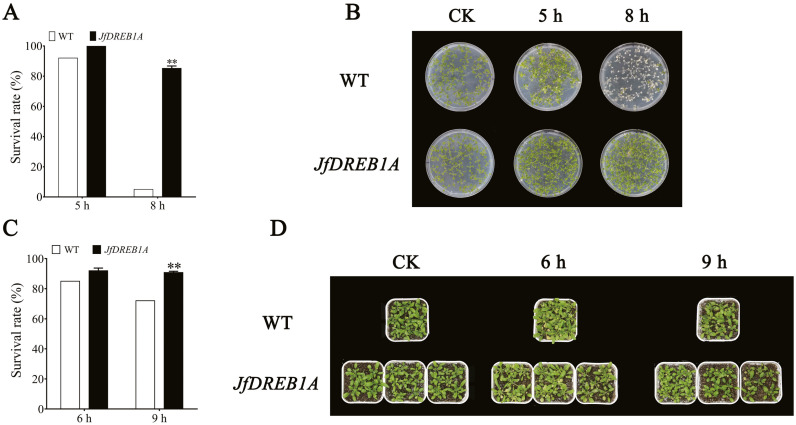
Overexpression of *JfDREB1A* enhanced tolerance of transgenic *Arabidopsis* under low temperature stress. (A) Survival rates of petri dish seedlings of transgenic and WT *Arabidopsis* under low temperature stress (−7 °C) for 5 h, 8 h. (B) Phenotypic changes of petri dish seedlings of transgenic and WT *Arabidopsis* under low temperature stress (−7 °C) for 5 h, 8 h. (C) Survival rates of potted seedlings of transgenic and WT *Arabidopsis* under low temperature stress (−7 °C) for 6 h, 9 h. (D) Phenotypic changes of potted seedlings of transgenic and WT *Arabidopsis* under low temperature stress (−7 °C) for 6 h, 9 h. WT, Wild type *Arabidopsis*; *JfDREB1A*, Transgenic *Arabidopsis* lines. The error bars indicate the SDs from three biological replicates. The asterisks above the bars indicate significant differences at the *P* < 0.05 level according to Duncan’s multiple comparison tests.

The transgenic and wild-type *Arabidopsis* seedlings were treated at −7 °C. The results showed that there were no significant differences in the phenotype between transgenic and wild-type *Arabidopsis* seedlings under normal growth conditions (23 °C) (Figs. 4B and 4D). After being exposed to the low-temperature stress at −7 °C for 5 h and a recovery period of 4 d, most of the leaves of petri dish seedlings were still green. The survival rates of transgenic and wild-type seedlings were 100% and 92%, respectively. Transgenic seedlings still maintained a high survival rate (85%) when the chilling stress extended to 8 h, while the leaves of wild-type seedlings were severely damaged by freezing with a survival rate of only 5% (Figs. 4A and 4B).

The potted seedlings showed similar results. The survival rate of transgenic seedlings was 91%, while wild-type seedlings was 72% after the low-temperature stress at −7 °C for 9 h (Figs. 4C and 4D). These results indicated that the overexpression of *JfDREB1A* improved the cold resistance of transgenic *Arabidopsis* seedlings.

### Overexpression of the *JfDREB1A* gene caused the physiological change in transgenic *Arabidopsis*

**Figure 5:**
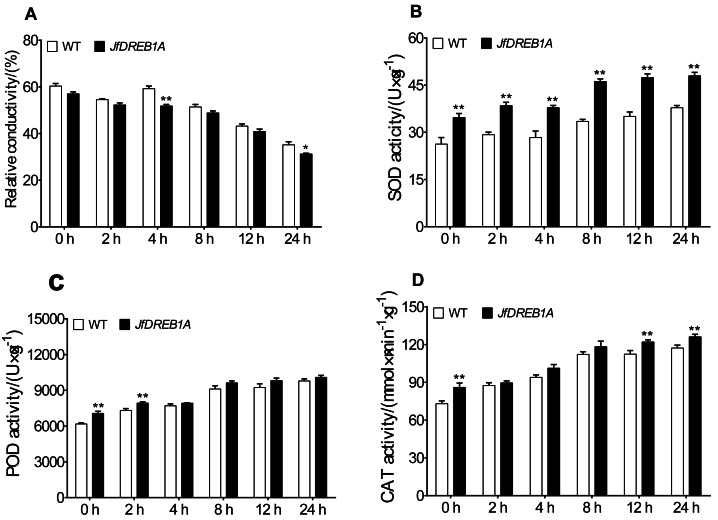
Changes in physiological parameters of transgenic and WT *Arabidopsis* under low temperature stress. (A–D) Relative electric conductivity, SOD, POD, and CAT activities were measured in transgenic and WT *Arabidopsis* seedlings under low temperature stress. WT, Wild type *Arabidopsis*; *JfDREB1A*, Transgenic *Arabidopsis* lines. The error bars indicate the SDs from three biological replicates. The asterisks above the bars indicate significant differences at the *P* < 0.05 level according to Duncan’s multiple comparison tests.

The relative electrical conductivity, SOD activity, POD activity, and CAT activity of transgenic and wild-type *Arabidopsis* seedlings were measured at 4 °C to analyze the effects of *JfDREB1A* gene overexpression on the physiological activities of plant cells. The results showed that the relative electrical conductivities gradually decreased and was lowest at 24 h and that transgenic *Arabidopsis* were always lower than the wild-type (Fig. 5A). Under low-temperature stress, the SOD activities, POD activities, and CAT activities of transgenic and wild-type *Arabidopsis* gradually increased, ascending most significantly at 8 h and reaching the highest levels at 24 h. The antioxidative enzyme activities of transgenic *Arabidopsis* were always higher than wild-type *Arabidopsis* under low-temperature stress (Figs. 5B–5D).

### Overexpression of the *JfDREB1A* gene increased the transcription level of cold-inducible genes

**Figure 6:**
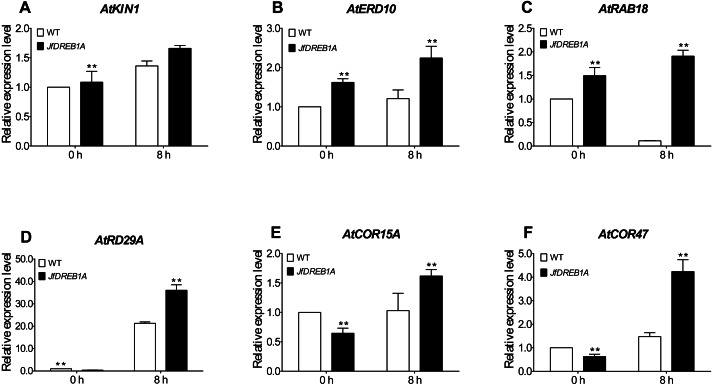
Expression levels of cold-inducible genes in transgenic and WT *Arabidopsis* under low temperature stress. (A) *AtKIN1* (NM_121601.2) gene, (B) *AtERD10* (NM_180616) gene, (C) *AtRAB18* (U75603.1) gene, (D) *AtRD29A* (D13044.1) gene, (E) *AtCOR15A* (NC_003071.7), (F) *AtCOR47* (NM_101894.4). The potted seedlings of transgenic and WT *Arabidopsis* were treated at 4 °C for 0 h (control), 8 h. The leaves were used for expression analysis, and the *AtUBQ3* gene was used for internal reference. WT, Wild type *Arabidopsis; JfDREB1A*, Transgenic *Arabidopsis* lines. The error bars indicate the SDs from three biological replicates. The asterisks above the bars indicate significant differences at the *P* < 0.05 level according to Duncan’s multiple comparison tests.

To further evaluate the involvement of the *JfDREB1A* gene to the cold stress response, we analyzed the expression levels of six cold-inducible genes which are related to low-temperature stress in *Arabidopsis*. The results showed that the expression levels of all genes were up-regulated except for *ATRAB18*. The *AtKIN1*, *AtERD10*, *AtRD29A*, *AtCOR15A* and *AtCOR47* genes were significantly higher than the control (0 h). The expression levels of all genes were higher in transgenic *Arabidopsis* than those in wild-type *Arabidopsis* (Fig. 6). These results indicated that the overexpression of the *JfDREB1A* gene improved the transcription activation ability of functional genes related to low-temperature stress.

## Discussion

Transcription factors regulate the expression of specific plant genes in response to abiotic stress such as low-temperature. *DREB1/CBF* can recognize CRT/DRE elements of the functional genes promoter region and plays an important role in the plant’s response to abiotic stress (Cook et al., 2004; Gilmour et al., 1998). The function of the *DREB1* gene in the ICE1(inducer of CBF expression 1)-CBF(C-repeat (CRT)-binding factor)-COR(cold responsive) cold signal pathway of plant had been reported extensively (Ding, Shi & Yang, 2019; Gilmour et al., 1998; Siddiqua & Nassuth, 2011; Stockinger, Gilmour & Thomashow, 1997). However, there are no reports on the function of DREB1 transcription factors in walnut, especially in the Xinjiang wild walnut.

In this study, the DREB members of walnut and *Arabidopsis* were mapped to draw a phylogenetic tree, and five DREB1 transcription factors of walnut were identified. These were named *DREB1A*, *DREB1B*, *DREB1C*, *DREB1Da,* and *DREB1Db* after being aligned with *Arabidopsis*. The expression levels showed that there were high levels of *DREB1A* in the root, stem, leaf. It was immediately up-regulated under low-temperature stress, and was found at significantly higher levels than the other four *DREB1* homologous genes. *DREB1A* can be regarded as a key gene involved in the regulation process of walnut to low-temperature stress.

**Figure 7:**
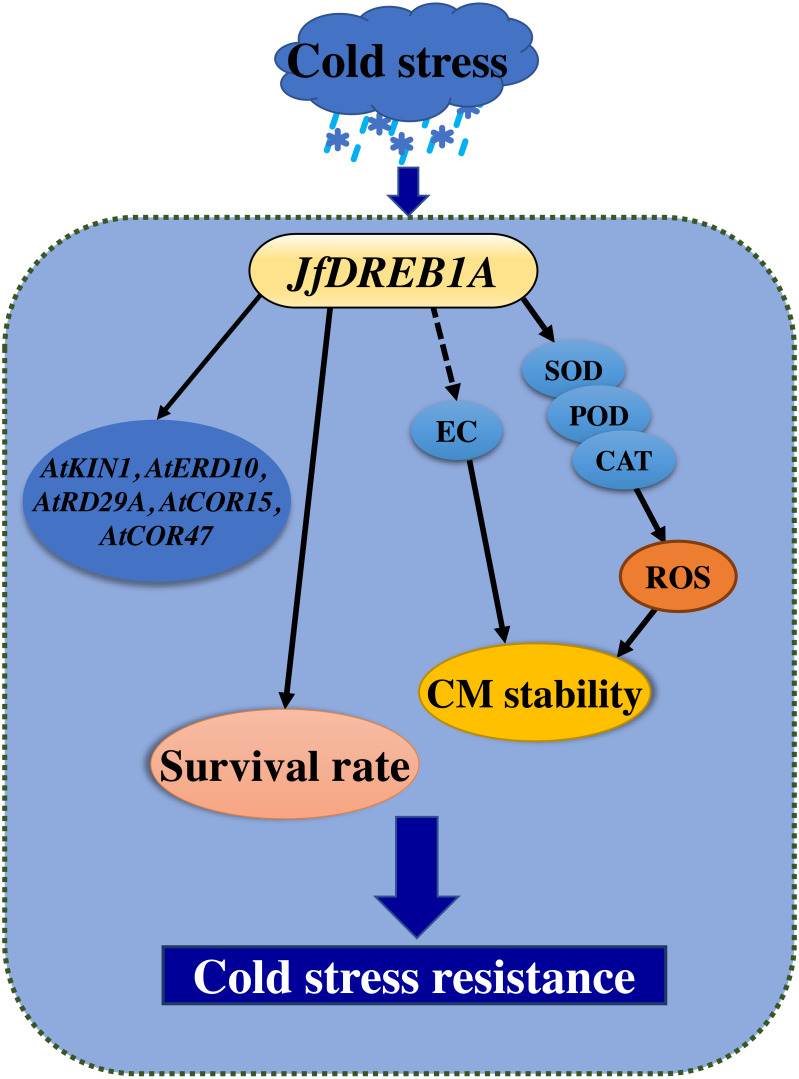
Cold stress model of transgenic seedlings regulated by overexpression of *JfDREB1A* gene. Solid lines indicate positive regulation, and dotted line indicate negative regulation. The overexpression of the *JfDREB1A* gene enhanced the survival rates of transgenic seedlings, decreased the relative electrical conductivities and increased SOD activities, POD activities, and CAT activities to maintain the stability of cell membrane, also positive regulated the expression of cold-induced genes. These results indicated that the expression of *JfDREB1A* gene could enhance the cold resistance of *Arabidopsis*.

Overexpression is one of the most common methods used to study gene function (Prelich, 2012). Previous studies have shown that the overexpression of the *DREB1A* gene can activate the expression of cold-inducible genes and promote the cold tolerance of transgenic plants (Gilmour et al., 2000; Hong et al., 2015; Maruyama et al., 2004; Oh et al., 2005; Shah et al., 2014). This study showed that the heterologous overexpression of the *JfDREB1A* gene improved the tolerance and increased the survival rates of transgenic *Arabidopsis* seedlings under low-temperature stress (Fig. 7).

A series of physiological responses occurred in plants under low-temperature stress. The cell membrane perceived the low-temperature stimulation, which led to the destruction of the cell structure and the loss of corresponding metabolic function (Gautier et al., 2013; Lyons, 1973). The stability of the plant cell membrane system is an important factor to consider when assessing the cold tolerance of plants (Muramatsu et al., 2015). The degree of damage to the cell membrane structure and function can be measured by its electrical conductivity (Liu et al., 2017). In this study, the relative electrical conductivities of transgenic seedlings were gradually decreased and were always lower than that of wild-type *Arabidopsis* under low-temperature stress (Fig. 7). The SOD activities, POD activities, and CAT activities of transgenic seedlings gradually increased and were always higher than wild-type *Arabidopsis* for 0–24 h at 4 °C (Fig. 7). Antioxidant enzymes are important in plants, and may eliminate redundant reactive oxygen species (ROS) to maintain the stability of the membrane system when their activity increases (Green & Fluhr, 1995). Therefore, the overexpression of *JfDREB1A* in transgenic *Arabidopsis* can reduce the contents of osmotic regulatory substances and improve the activities of antioxidant enzymes, thereby regulating the tolerance of plants to low-temperature stress.

The DREB1 protein binds to the CRT/DRE elements of the target cryogenic response genes to induce the expression levels of genes which respond to low-temperature (Shi, Ding & Yang, 2018). At present, CRT/DRE elements have been found in the cold stress-inducible genes *AtKIN1*, *AtERD10*, *AtRAB18*, *AtRD29A*, *AtCOR15A,* and *AtCOR47*, which are regulated by the DREB1 protein in *Arabidopsis* (Aleynova et al., 2020; Ding, Shi & Yang, 2019; Stockinger, Gilmour & Thomashow, 1997). We determined the expression of the above genes to further understand the activation of the *JfDREB1A* gene to downstream functional genes in transgenic *Arabidopsis*. The expression levels of *AtKIN1*, *AtERD10*, *AtRD29A*, *AtCOR15A* and *AtCOR47* were significantly increased under low-temperature stress (Fig. 7). These may be the direct target genes of *JfDREB1A*, however, further experiments are needed for verification.

## Conclusions

In this work, five *DREB1* genes were identified in the walnut. Among them, the relative expression levels of the *DREB1A* gene were high in the root, stem, and leaf and were immediately up-regulated under low-temperature stress. Its levels were significantly higher than the other four *DREB1* homologous genes. The *JfDREB1A* gene of Xinjiang wild walnut was cloned. The overexpression of the *JfDREB1A* gene in *Arabidopsis* was shown to enhance cold resistance in transgenic seedlings and improve the response to low-temperature stress, as well as positively regulate the expression of cold-induced genes. These results indicated that the expression of the *JfDREB1A* gene may enhance the cold resistance of *Arabidopsis*, which may play an important role in the response to cold stress of the Xinjiang wild walnut.

##  Supplemental Information

10.7717/peerj.14021/supp-1 Figure S1 Multiple sequence alignment of the 61 protein sequences of the DREB subfamily in walnut The 61 protein sequences all contain an AP2 domain with about 60 amino acids, among them the *DREB1* genes with yellow background color.

10.7717/peerj.14021/supp-2 Figure S2 Electrophoretic analyses of PCR products for *JfDREB1A* gene The *DREB1A* gene was cloned from Xinjiang wild walnut and named *JfDREB1A*, with a coding region for 645 bp through sequencing. (M) DL2000 marker. (1) Full length of *JfDREB1A* gene.

10.7717/peerj.14021/supp-3 Figure S3 Schematic diagram of JfDREB1A protein structure The protein consists of 214 amino acids, including an AP2 domain about 60 amino acids, with the structure features of the DREB1/CBF transcriptional factors.

10.7717/peerj.14021/supp-4 Figure S4 Expression levels of *JfDREB1A* gene in T3 generation transgenic and WT *Arabidopsis.* The open reading frame (ORF) of *JfDREB1A* gene was constructed into pCAMBIA1304 vector. The recombinant plasmid was transformed into *Agrobacterium tumefaciens* GV3101 strain by freezing-thawed and then transferred into *Arabidopsis* through the *Agrobacterium tumefaciens* soaking. After hygromycin screening and PCR detection, it was preliminarily proved that the *Arabidopsis* transformed with *JfDREB1A* gene was obtained. WT: Wild type *Arabidopsis*; *JfDREB1A*: Transgenic *Arabidopsis* lines. The error bars indicate the SDs from three biological replicates.

10.7717/peerj.14021/supp-5 Table S1 List of the primers used in this study

10.7717/peerj.14021/supp-6 Table S2 Classification of AP2 transcription factors in walnut and *Arabidopsis thaliana.*

10.7717/peerj.14021/supp-7 Table S3 DREB transcription factors in walnut and *Arabidopsis thaliana.*

10.7717/peerj.14021/supp-8 Table S4 The DNA sequence of the *JfDREB1A* gene The *JfDREB1A* gene have a coding region for 645 bp and marked in red.

10.7717/peerj.14021/supp-9 Data S1 Raw data

## Additional Information and Declarations

### Data Availability

The following information was supplied regarding data availability: The raw data is available in the Supplemental Files.
