# Supplementary figures and images for: The cold-stress responsive gene DREB1A involved in low-temperature tolerance in Xinjiang wild walnut
Source: PeerJ. 2022 Sep 8;10:e14021. doi: 10.7717/peerj.14021 (PMC9464435; doi:10.7717/peerj.14021)

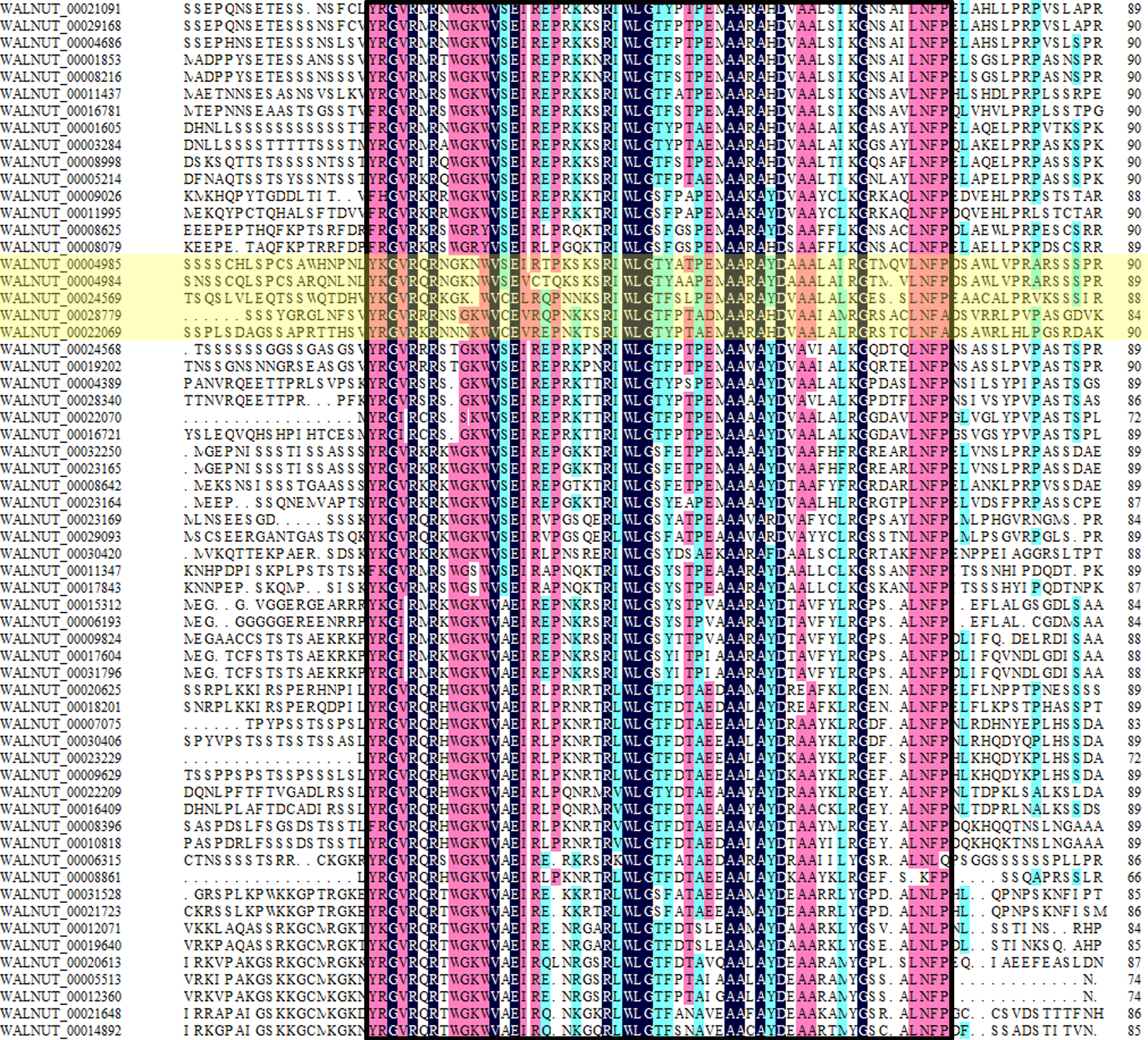

Supplement: Figure S1 — The 61 protein sequences all contain an AP2 domain with about 60 amino acids, among them the DREB1 genes with yellow background color. [file peerj-10-14021-s001.png]

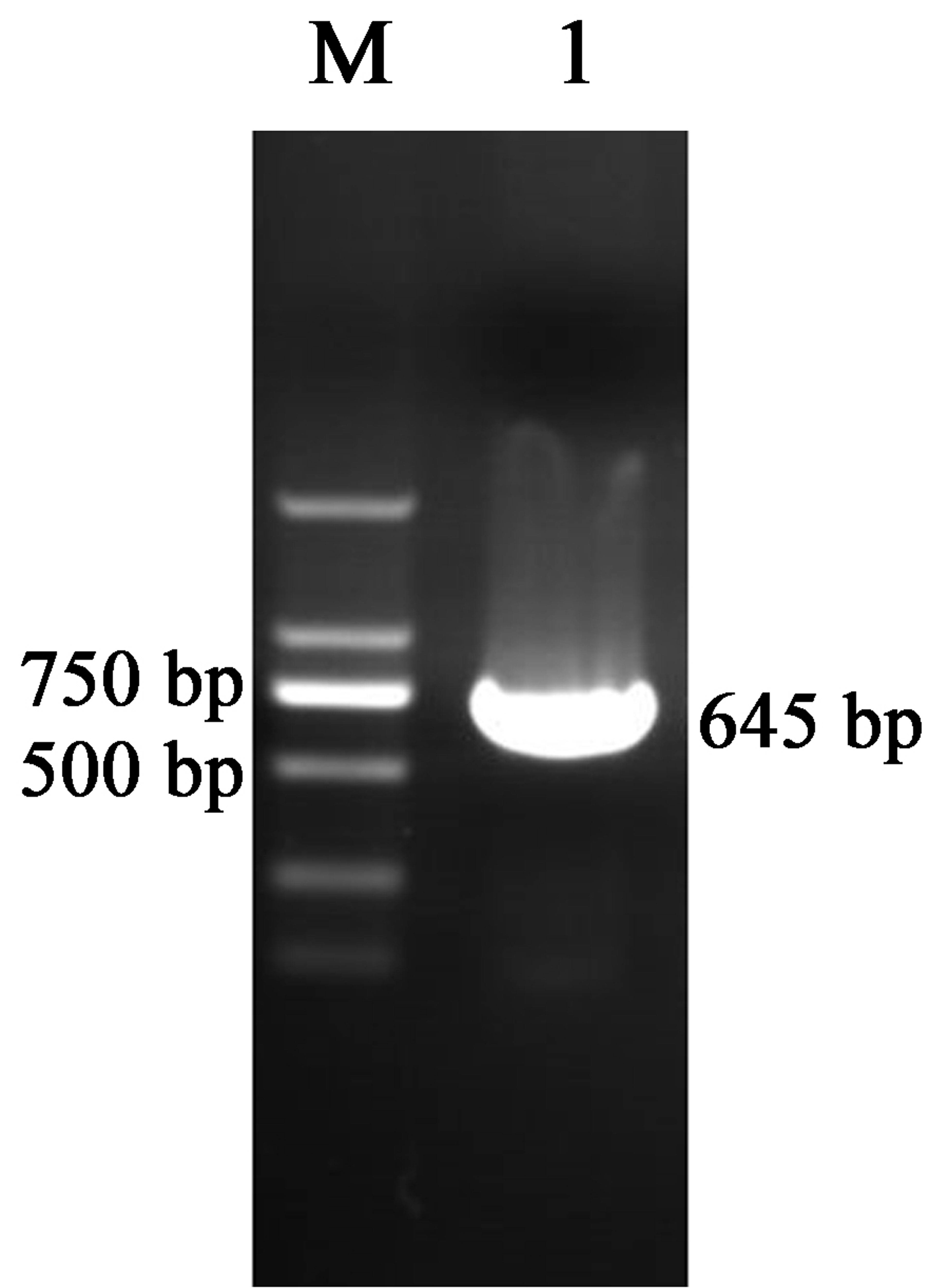

Supplement: Figure S2 — The DREB1A gene was cloned from Xinjiang wild walnut and named JfDREB1A, with a coding region for 645 bp through sequencing. (M) DL2000 marker. (1) Full length of JfDREB1A gene. [file peerj-10-14021-s002.png]

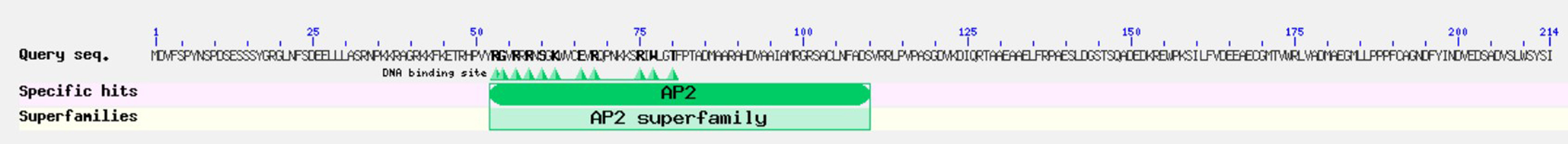

Supplement: Figure S3 — The protein consists of 214 amino acids, including an AP2 domain about 60 amino acids, with the structure features of the DREB1/CBF transcriptional factors. [file peerj-10-14021-s003.png]

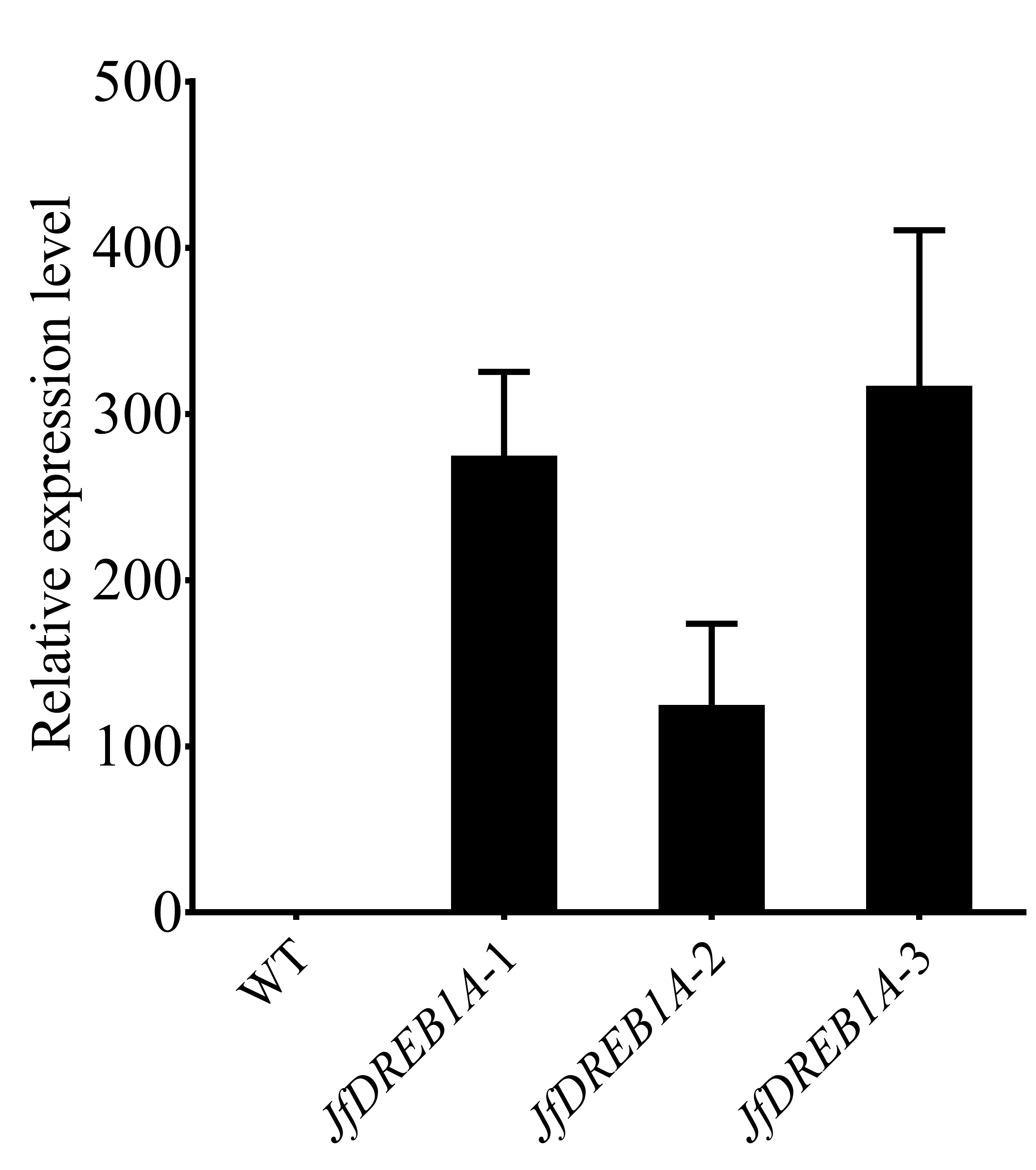

Supplement: Figure S4 — The open reading frame (ORF) of JfDREB1A gene was constructed into pCAMBIA1304 vector. The recombinant plasmid was transformed into Agrobacterium tumefaciens GV3101 strain by freezing-thawed and then transferred into Arabidopsis through the Agrobacterium tumefaciens soaking. After hygromycin screening and PCR detection, it was preliminarily proved that the Arabidopsis transformed with JfDREB1A gene was obtained. WT: Wild type Arabidopsis; JfDREB1A: Transgenic Arabidopsis lines. The error bars indicate the SDs from three biological replicates. [file peerj-10-14021-s004.png]
